# Tuning the Magnetic Response of Magnetospirillum magneticum by Changing the Culture Medium: A Straightforward Approach to Improve Their Hyperthermia Efficiency

**DOI:** 10.1021/acsami.2c18435

**Published:** 2022-12-23

**Authors:** David Gandia, Lourdes Marcano, Lucía Gandarias, Danny Villanueva, Iñaki Orue, Radu Marius Abrudan, Sergio Valencia, Irati Rodrigo, José Ángel García, Alicia Muela, Ma Luisa Fdez-Gubieda, Javier Alonso

**Affiliations:** † Basque Center for Materials Applications and Nanostructures (BCMaterials) UPV/EHU Science Park Leioa, Leioa48940, Spain; ‡ Departmento de Física, Facultad de Ciencias, Universidad de Oviedo, Oviedo33007, Spain; § Departamento de Inmunología, Microbiología y Parasitología, Universidad del País Vasco (UPV/EHU), Leioa48940, Spain; ∥ Departamento de Electricidad y Electrónica, Universidad del País Vasco (UPV/EHU), Leioa48940, Spain; ⊥ SGIker Medidas Magnéticas, Universidad del País Vasco (UPV/EHU), Leioa48940, Spain; # Helmholtz-Zentrum Berlin für Materialien und Energie, Albert-Einstein-Street 15, Berlin12489, Germany; ¶ Departamento Física Aplicada, Universidad del País Vasco (UPV/EHU), Eibar20600, Spain; ∇ Departamento Física Aplicada, Universidad del País Vasco (UPV/EHU), Leioa48940, Spain; ○ Departamento CITIMAC, Universidad de Cantabria, Santander39005, Spain

**Keywords:** magnetotactic bacteria, magnetic hyperthermia, culture medium, magnetosomes, magnetic properties, simulations

## Abstract

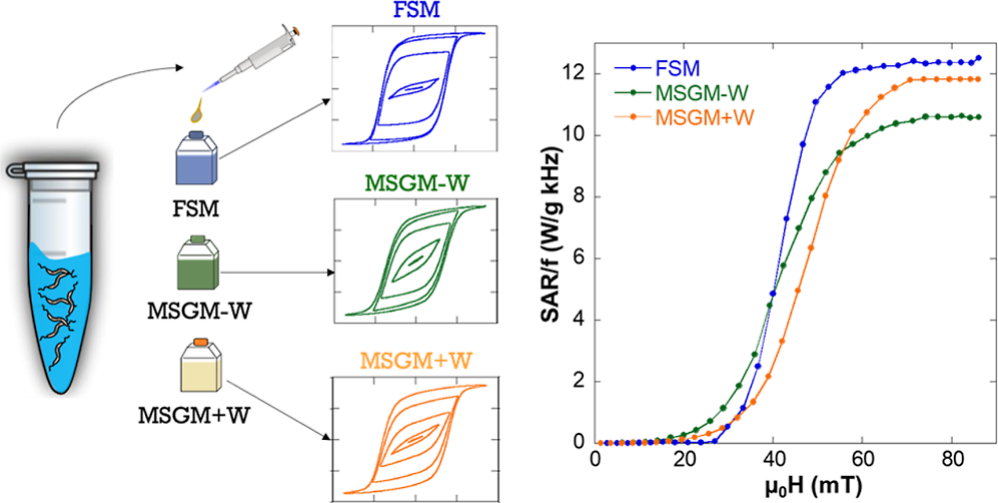

Magnetotactic bacteria *Magnetospirillum magneticum* AMB-1 have been cultured using three different media: magnetic spirillum growth medium with Wolfe’s mineral solution (MSGM + W), magnetic spirillum growth medium without Wolfe’s mineral solution (MSGM – W), and flask standard medium (FSM). The influence of the culture medium on the structural, morphological, and magnetic characteristics of the magnetosome chains biosynthesized by these bacteria has been investigated by using transmission electron microscopy, X-ray absorption spectroscopy, and X-ray magnetic circular dichroism. All bacteria exhibit similar average size for magnetosomes, 40–45 nm, but FSM bacteria present slightly longer subchains. In MSGM + W bacteria, Co^2+^ ions present in the medium substitute Fe^2+^ ions in octahedral positions with a total Co doping around 4–5%. In addition, the magnetic response of these bacteria has been thoroughly studied as functions of both the temperature and the applied magnetic field. While MSGM – W and FSM bacteria exhibit similar magnetic behavior, in the case of MSGM + W, the incorporation of the Co ions affects the magnetic response, in particular suppressing the Verwey (∼105 K) and low temperature (∼40 K) transitions and increasing the coercivity and remanence. Moreover, simulations based on a Stoner–Wolhfarth model have allowed us to reproduce the experimentally obtained magnetization versus magnetic field loops, revealing clear changes in different anisotropy contributions for these bacteria depending on the employed culture medium. Finally, we have related how these magnetic changes affect their heating efficiency by using AC magnetometric measurements. The obtained AC hysteresis loops, measured with an AC magnetic field amplitude of up to 90 mT and a frequency, *f*, of 149 kHz, reveal the influence of the culture medium on the heating properties of these bacteria: below 35 mT, MSGM – W bacteria are the best heating mediators, but above 60 mT, FSM and MSGM + W bacteria give the best heating results, reaching a maximum heating efficiency or specific absorption rate (SAR) of SAR/*f* ≈ 12 W g^–1^ kHz^–1^.

## Introduction

Magnetotactic bacteria (MTB) are aquatic motile microorganisms with the ability to align and orient themselves in the presence of Earth’s magnetic field.^1^ This special property arises due to the presence of one or several chains of magnetic nanoparticles coated with a lipid bilayer membrane, called magnetosomes.^2,3^ The mineral core of these magnetosomes presents high chemical purity, being made of magnetite, Fe_3_O_4_, in most of the species, though some of them synthesize greigite, Fe_3_S_4_. The size of the magnetic crystals typically ranges between 35 and 120 nm, being magnetically stable at room temperature.^4,5^ Magnetosomes also exhibit high crystallinity, biocompatibility, and magnetic response, thereby attracting great interest in different research areas, especially those related to cancer treatment and similar biomedical applications.^6−12^ In addition, magnetosomes, due to their exceptional characteristics, have also been considered as ideal model systems of magnetic nanoparticles.^13−21^

In recent years, there has been growing interest in using not only the isolated magnetosomes but also the whole MTB as biomedical nanobio-robots, *nanobiots*, for different theranostic applications such as magnetic resonance imaging, magnetic particle imaging, drug delivery, magnetic hyperthermia, and so forth.^8,11,12,22−26^ Seizing the advantage of the self-propulsion capability provided by their flagella and the presence of the magnetosome chain, these bacteria can be guided and manipulated by external magnetic fields toward specific areas inside the human body. On top of that, MTB, due to their preference for low oxygen concentration environments, are naturally attracted toward hypoxic areas, such as tumor regions.^8^ In addition, it has been shown that MTB can be efficiently internalized by cancer cells, exhibiting very low cytotoxicity.^11^

One of the main parameters that determines the magnetic response of magnetic nanoparticles in general, and MTB in particular, for biomedical applications is the effective magnetic anisotropy.^27−30^ The effective magnetic anisotropy depends on several contributions such as the surface, shape, crystallinity, and so forth,^31^ and it has been shown that tailoring the magnetic anisotropy of the nanoparticles can greatly increase, for example, the heating efficiency of the nanoparticles in magnetic hyperthermia treatment.^27,32−37^ In this respect, MTB have already proven to be exceptional heating mediators.^11^ However, MTB also present some limitations. It is well known that the structure of the magnetosome chain and the morphology and size of the magnetosomes are genetically controlled and specific to each species of bacteria.^4,38^ Therefore, to obtain, for example, magnetosomes with different morphologies, one would have to use a different species of MTB. In this respect, although it is true that there are many species of MTB that biomineralize magnetosomes with different morphologies (e.g., cube-octahedral, elongated prismatic, bullet- or tooth-shaped), most of these species are fastidious to culture and grow in the laboratory. Therefore, most of the research works have been focused on only a few of them, those that have consistently exhibited cell growth and full-chain biomineralization under controlled conditions.^39−41^ Among these “more reliable” species of MTB, we can find *Magnetospirillum magneticum* AMB-1. This is a freshwater species of MTB with spirillum morphology, which synthesizes truncated-octahedral magnetosomes with a slight distortion and a mean size of ∼40 nm. Contrary to other similar spirillum species, such as *Magnetospirillum gryphiswaldense* MSR-1, AMB-1 synthesizes fragmental chains instead of a long continuous chain.^42^ The latter species has already been investigated in several biomedical applications, including magnetic hyperthermia.^43^ There have been however only a few works which analyze the magnetic response of this species of MTB, and the number of works which attempt to modify this magnetic response is even more limited.^42,44−46^ One route to modify the magnetic response of this and other species of MTB is by changing the culture medium and growth conditions.^44,45,47−49^ According to the literature, *M. magneticum* AMB-1 is predominantly grown using a magnetic spirillum growth medium with a supplement of Wolfe’s mineral solution.^50−52^ Under these conditions, it has been consistently reported that the growth and biomineralization process of this species are optimized. However, there is an essential issue with this growth medium that has been ignored so far: it contains low concentrations of transition-metal chlorides and sulfates, such as MnSO_4_, FeSO_4_, CoCl_2_, ZnSO_4_, or CuSO_4_. It is worth noting that *M. magneticum* has already demonstrated the ability to incorporate transition metals into magnetosomes.^45,53^ Therefore, it is likely that the magnetosomes grown under these “ideal conditions” are not purely made of magnetite but doped with some additional transition-metal ions incorporated into their structure which might modify their magnetic properties. To shed light onto this, we have grown *M. magneticum* AMB-1 using a revised magnetic spirillum growth medium with (MSGM + W) and without (MSGM – W) Wolfe’s mineral solution. Finally, we have also employed a third different medium for growing this species, flask standard medium (FSM).^54^ This growth medium is simpler than MSGM in its elaboration but rarely used for this species. By using three different media, we expect to modify the magnetic response of *M. magneticum* AMB-1 in a simple and straightforward way, with the aim of improving their efficiency in biomedical applications such as magnetic hyperthermia.

Therefore, in this work, we have investigated the influence of the culture medium on the magnetic response of *M. magneticum* AMB-1 using three different media: MSGM + W, MSGM – W, and FSM. The structural and morphological changes of the magnetosome chains have been investigated by using transmission electron microscopy (TEM). The incorporation of additional metal ions into the magnetosome structure has been analyzed by inductively coupling plasma–atomic emission spectroscopy (ICP–AES), X-ray absorption spectroscopy (XAS), and X-ray magnetic circular dichroism (XMCD) experiments carried out in a large-scale synchrotron facility. In addition, the magnetic response of AMB-1 bacteria grown in these three media has been compared by using a combination of different magnetic measurements, including zero-field cooling/field-cooling (ZFC/FC) curves and hysteresis loops (*M* vs *H*). A modified Stoner–Wohlfarth model has been employed to simulate the experimental *M* versus *H* loops. This has allowed us to better pinpoint the specific magnetic changes taking place in these bacteria depending on the culture medium. Finally, we have tested how these bacteria grown in different media respond under magnetic hyperthermia conditions using AC magnetometry methods.^55−57^ In these measurement, the AC hysteresis loops described by the magnetic moments of the magnetosomes, under a certain AC magnetic field of frequency *f*, are measured, and the heating efficiency is directly obtained from the area of these AC hysteresis loops. These measurements have provided us a clear depiction on how the heating efficiency, also called the specific absorption rate or SAR, of these bacteria is modified depending on the culture medium. Our results indicate that above 60–65 mT, the SAR tends to saturate for all samples, reaching a maximum value SAR/*f* ≈ 12 W g^–1^ kHz^–1^ for FSM and MSGM + W bacteria, which coincidentally exhibit the highest uniaxial anisotropy (*K*_uni_ = 16(4) kJ/m^3^). However, for MSGM – W bacteria with a lower uniaxial anisotropy (*K*_uni_ = 12(4) kJ/m^3^), the maximum SAR/*f* value reached reduces to ∼10.5 W g^–1^ kHz^–1^.

## Experimental Techniques

### Magnetotactic Bacterial Culture

*M. magneticum* AMB-1 was grown in 1 L bottles, closed to achieve microaerophilic conditions at 28 °C without shaking, and filled with three different media: MSGM + W, MSGM – W, and FSM.

For MSGM + W/–W, the cells were grown in a medium containing (per liter of deionized water) 0.035 g L-ascorbic acid 0.07 M sodium acetate, 0.1 g sodium thiosulfate, 0.12 g NaNO_3_, 0.37 g succinic acid, 0.37 g l-tartaric acid, and 0.68 g KH_2_PO_4_. For magnetosome formation, 10 mL of iron(III) citrate (10 mM) was added per liter of culture media. In addition, in the case of MSGM + W, 5 mL of Wolfe’s mineral solution was added to the medium, which contained 3 g MgSO_4_·7H_2_O, 0.5 g MnSO_4_·H_2_O, 0.1 g NaCl, 0.1 g CoCl_2_·6H_2_O, 0.13 g CaCl_2_·2H_2_O, 0.1 g ZnSO_4_·7H_2_O, 0.01 g CuSO_4_·5H_2_O, 0.01 g AlK(SO)_4_·12H_2_O, 0.01 g H_3_BO_3_, and 0.01 g Na_2_MoO_4_·2H_2_O.^53^ A magnetic inoculum with cells at the early stationary phase was employed. Cells were collected after 48 h of incubation, when bacteria present well-formed magnetosomes chains.

For FSM, the cells were grown in a medium containing (per liter of deionized water) 0.1 g KH_2_PO_4_, 0.15 g MgSO_4_·7H_2_O, 2.38 g HEPES, 0.34 g NaNO_3_, 0.1 g yeast extract, 3 g soybean peptone, 0.3% (wt/vol) of sodium pyruvate as the carbon source, and 100 μM Fe(III) citrate. In this case, the cells were collected after 96 h of incubation to assure the presence of well-formed magnetosome chains.

The collected cells were fixed with 2% glutaraldehyde, harvested by centrifugation, washed three times, and finally concentrated up to ∼10^11^ cells/mL in ultrapure water.

### Transmission Electron Microscopy

TEM was performed on unstained cells adsorbed onto 300 mesh carbon-coated copper grids. TEM images were obtained with a JEOL JEM-1400 Plus electron microscope at an accelerating voltage of 120 kV. The particle size distribution was analyzed using a standard software for digital electron microscope image processing, ImageJ.^58^

### Inductively Coupling Plasma Atomic Emission Spectroscopy

The Fe, Co, Mn, Cu, and Zn contents of the three samples were measured by ICP-AES technique, using an Agilent 5100 spectrophotometer. 100 μL concentrated bacterial samples (∼10^11^ cells/mL) were each digested at 80 °C in 300 μL of nitric acid. The solutions were diluted 50 times in Milli-Q water.

### X-ray Magnetic Circular Dichroism

XMCD experiments were carried out using the ALICE station at the PM3 beamline of BESSY II in Berlin, Germany. All XAS spectra were collected at room temperature. A drop of 5 μL of bacteria in aqueous solution with a concentration of ∼10^11^ cells/mL was deposited onto carbon-coated copper grids (similar to those used for TEM). Data acquisition was done in the transmission mode with the incoming circularly polarized (right helicity) X-rays impinging at normal incidence with respect to the sample surface. A magnetic field of ±0.35 T was applied along the beam propagation direction. XAS spectra (*I*) were obtained across the Mn, Fe, and Co L_2,3_-edges with a step size of 0.2 eV with an applied magnetic field parallel to the X-ray beam of +0.35 T (*I*^+^) and −0.35 T (*I*^–^). XMCD was computed as *I*^+^ – *I*^–^.

### Magnetic Measurements

For the magnetic measurements, the bacteria were freeze-dried obtaining a powder sample. This powder, containing the bacteria randomly oriented, was inserted inside a transparent gelatin capsule, and pressed inside the capsule to avoid any movement of the powder during the measurement. Finally, the gelatin capsule was inserted into a plastic transparent straw, placed at the end of the sample holder rod, and inserted into the magnetometer for the magnetic measurements. Magnetic measurements were performed in a superconducting quantum interference device magnetometer (Quantum Design MPMS-3). Magnetization versus temperature (*M* vs. *T*) curves were measured following the usual ZFC/FC protocol, with an applied magnetic field of 5 mT.

**Figure 1:**
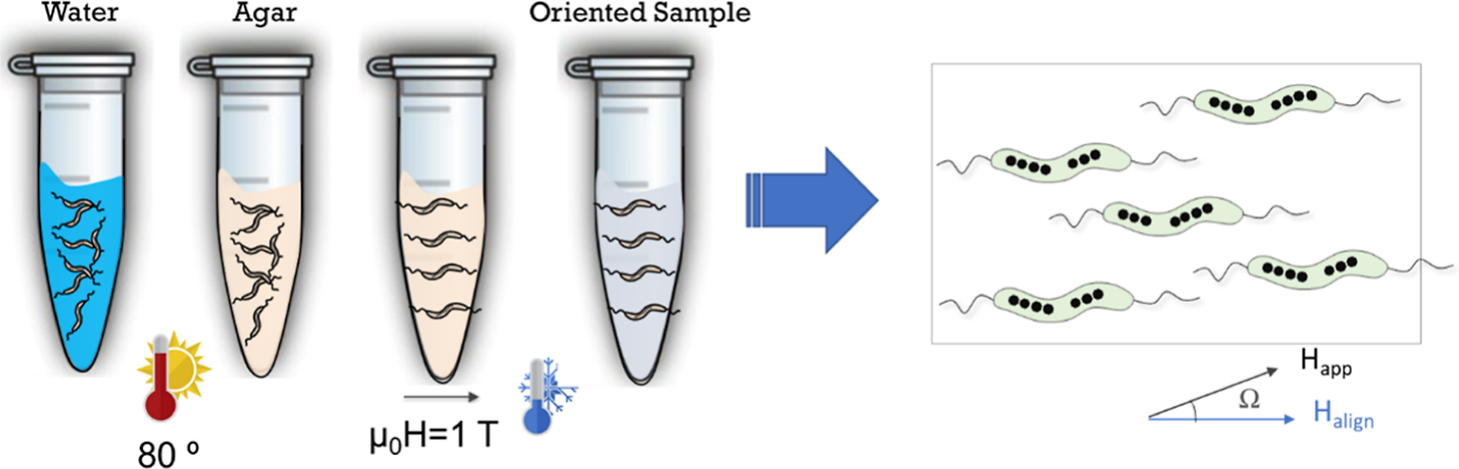
Schematic representation of the process carried out to obtain aligned MTB in agar, as described in the text. In the *M* versus *H* hysteresis loops measured at room temperature the external applied field, *H*_app_, forms a variable angle Ω with respect to the alignment field, *H*_align._

Macroscopically oriented hysteresis loops (*M* vs *H*) at room temperature were measured by vibrating sample magnetometry (VSM). 3D arrangements of aligned bacteria were obtained by resuspending 500 μL of a bacterial colloid (∼10^11^ cell/mL) in 500 μL of an agar solution composed of 2% agar and 98% water at 80 °C to maintain the solution in a liquid state. To align the bacteria, a uniform magnetic field of 1 T was applied. After 3 min the sample was cooled using liquid nitrogen until the temperature reached around 0 °C, and the field was turned off. This caused the agar to solidify, trapping the bacteria, and keeping this solidified state at room temperature, as it is explained in Figure 1.

### Magnetic Hyperthermia

Magnetic hyperthermia studies have been performed using AC magnetometry methods on randomly oriented bacteria. A homemade setup^57^ was employed to record the AC hysteresis loops. The AC magnetic field amplitude was tuned between 0 and 90 mT, being the frequency 149 kHz. For these measurements, we prepared suspensions of bacteria in distilled water with a cell concentration around ∼10^11^ cell/mL for all studied samples. The volume of the sample vial was 100 μL. The total magnetite concentration was 0.42, 0.63, and 0.17 mg Fe_3_O_4_ mL^–1^ for MSGM – W, MSGM + W, and FSM bacteria, respectively.

## Results and Discussion

### Morphological and Structural Characterizations

**Figure 2:**
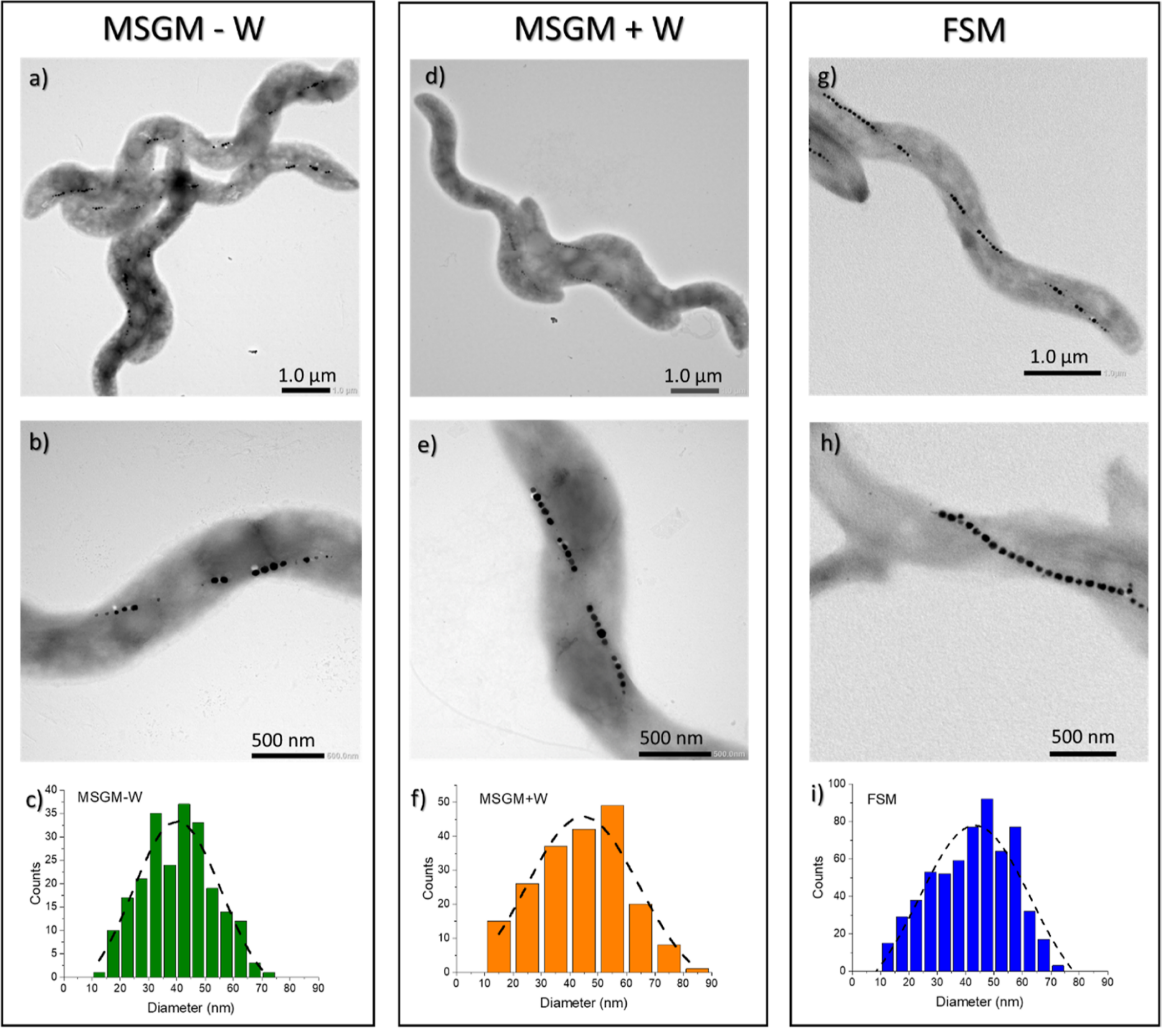
Representative TEM images of *M. magneticum* grown in MSGM – W, MSGM + W, and FSM: (a,d,g) general depiction of the whole cells, (b,e,h) focus on the magnetosome chains, and (c,f,i) size distribution histograms for the magnetosomes inside the bacteria, together with the Gaussian fittings.

Figure 2 presents the TEM images of *M. magneticum* grown in MSGM – W, MSGM + W, and FSM. As depicted, bacteria in the three tested conditions present the spirillum shape and fragmented chains typical of this species. We have compared the number of subchains per cell (*N*_sub_), the number of magnetosomes per subchain (*N*_mag_), and the size distribution of the magnetosomes including their average size (*D*) and standard deviation (σ), as indicated in Table 1. The three samples present a similar number of subchains per cell (*N*_sub_), around 4 or 5. However, although MSGM – W and MSGM + W exhibit quite uniform subchains, with an average number (*N*_mag_) of 5 magnetosomes per subchain, in the case of FSM, these subchains are far less homogeneous, with a *N*_mag_ widely varying from 4 to 12. Finally, some differences can also be observed in the size of the magnetosomes. For the three samples, the average sizes (*D*) are similar, albeit slightly smaller for MSGM – W. Concerning the morphology of the magnetosomes, in principle no relevant differences are observed between the *M. magneticum* grown in different media, although high-resolution TEM and/or tomography images would be needed for a better comparison.

**Table 1:** Analysis of TEM Images Obtained for MSGM – W, MSGM + W, and FSM Bacteria^a^

	*N*_sub_	*N*_mag_	*D* (nm)	σ
MSGM – W	4(1)	5(1)	40(1)	15(4)
MSGM + W	5(1)	5(1)	44(2)	16(5)
FSM	4(1)	8(4)	43(1)	19(6)

a *N*_sub_ is the average number of subchains per cell, *N*_mag_ corresponds to the average number of magnetosomes per subchain, and *D* and σ are the average size and standard deviation of the magnetosomes, respectively, as obtained from the size distribution histograms. Around 200–250 magnetosomes were analyzed in this study.

As mentioned before, *M. magneticum* has already demonstrated the capacity to incorporate metals into the magnetosomes. Therefore, we wanted to test if the MSGM + W bacteria, grown in a medium (Wolfe’s mineral solution) that contains different metal chlorides and sulphates, also incorporated any of these elements into the magnetite structure. For this, as a preliminary analysis, we tested by means of ICP-AES whether bacteria grown in MSGM + W incorporate the metals contained in the Wolfe solution. Among these metals, we focused on those that could more easily be incorporated into the bacteria (Co, Mn, Zn, Cu).^49^ These results are compared to those of bacteria grown in MSGM – W and in FSM in Table 2. We must remark that ICP–AES has been performed on the whole bacteria, and though it can detect the presence of these elements, their exact location is unknown, that is, they could be incorporated not only in magnetosomes but also in different cell compartments of bacteria. The presence of Co is only detected in MSGM + W bacteria, while Mn, Fe, Cu, and Zn are detected in all samples. The amount of Mn, Cu, and Zn normalized to the amount of Fe in the samples is very similar for both MSGM – W and FSM. However, for MSGM + W, these elements are present in a higher quantity. These data evidence that bacteria grown in MSGM + W do incorporate these metals in a larger amount than bacteria grown in MSGM – W and FSM. However, note that the incorporation of the metals does not imply that these are in the magnetite core of the magnetosomes, as they could be stored in other bacterial compartments, as reported for MSR-1 species.^49^ Indeed, from previous studies, the elements that most likely can be introduced into the magnetite structure are Co and Mn.^48,59^

**Table 2:** Mass of Elements Present in the Bacteria Normalized by the Total Mass of Fe in the Three Samples, as Measured by ICP–AES

	MSGM – W	MSGM + W	FSM
Mn/Fe	0.0011(6)	0.0064(3)	0.002(2)
Co/Fe	0.0006(6)	0.0016(3)	0.002(2)
Cu/Fe	0.0067(6)	0.0431(4)	0.007(2)
Zn/Fe	0.006(1)	0.0116(8)	0.009(4)

To verify whether bacteria grown in MSGM + W incorporate Co and Mn into the magnetite structure of the magnetosomes, we performed synchrotron XAS and XMCD experiments on the MSGM + W bacteria at Mn, Fe, and Co absorption L_2,3_–edges. Both XAS and XMCD are very powerful element-sensitive techniques that provide complementary and very valuable information as detailed in the following. XAS has allowed us to verify the incorporation of the different doping elements (Co and Mn) into the bacteria, either in the magnetosomes or in other bacterial compartments. On the other hand, XMCD, being a technique sensitive to the magnetic response, has allowed us to obtain accurate information on the oxidation state and site occupancy of these ions in the spinel structure of the magnetite core of magnetosomes.

**Figure 3:**
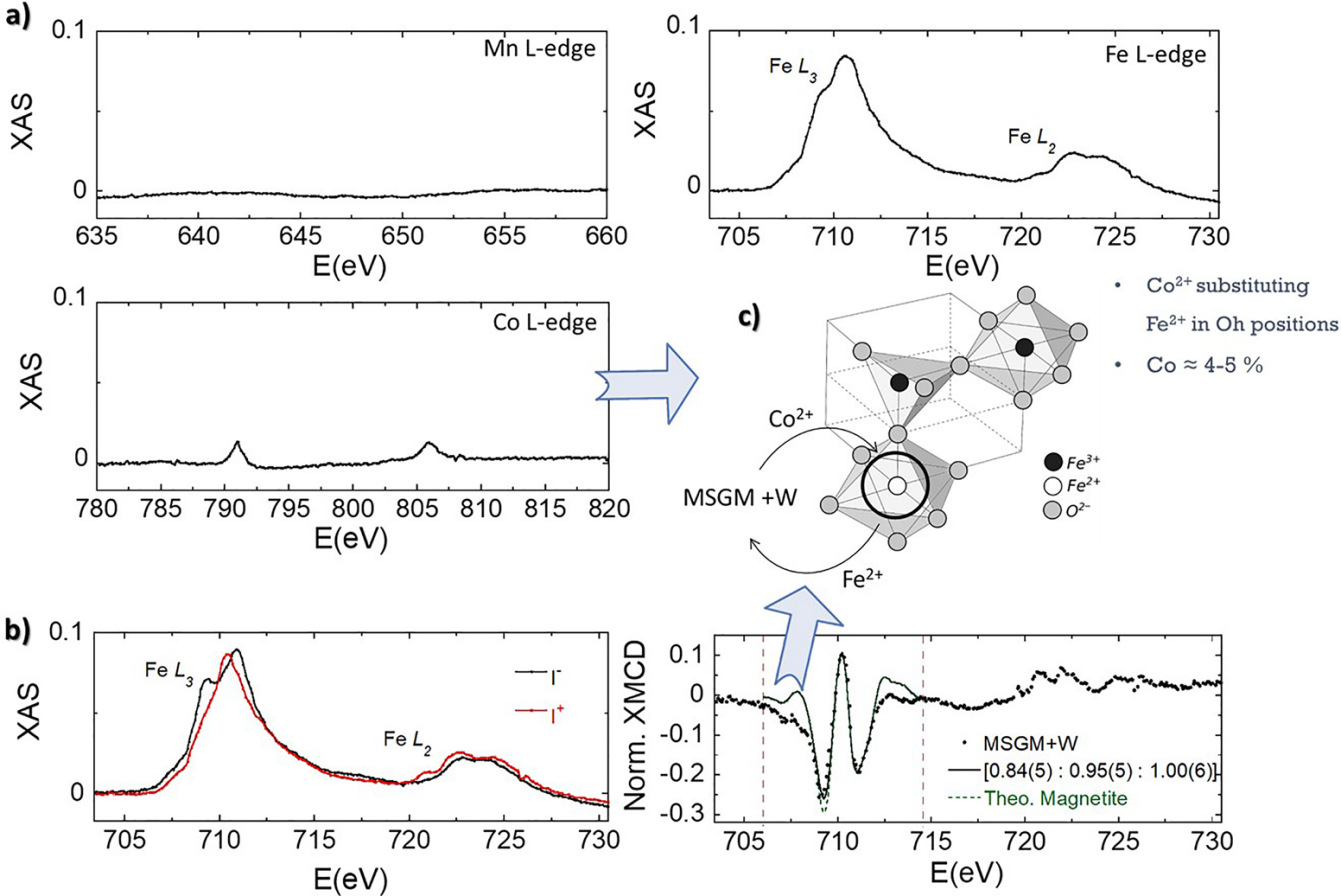
(a) Nonmagnetic contribution of XAS spectra for MSGM + W, (*I*^+^ + *I*^–^)/2, measured at Fe, Mn, and Co L-edges in the transmission mode at 300 K. XAS signal obtained for MSGM + W measured at Fe L-edge. (b) Computing *I*^+^ – *I*^–^ gives the XMCD signal. Spectra have been normalized by the peak intensity at the L_3_-edge of nonmagnetic XAS. The continuous line presents the best theoretical simulation of the Fe L_3_-edge XMCD spectra in the energy window delimited by the vertical dotted lines. The theoretical spectrum of magnetite has been superimposed for comparison. (c) Schematic depiction of the incorporation of Co^2+^ ions into the magnetosome structure for MSGM + W.

Figure 3a shows the room temperature nonmagnetic X-ray absorption, obtained from the semisum (*I*^+^ + *I*^–^)/2, with a magnetic field of ±0.35 T (*I*^+^/*I*^–^), at the Mn, Fe, and Co L_2,3_-edges. As can be seen, only those spectra measured at the Fe and Co edges exhibit absorption peaks. This already indicates that, if Mn ions are incorporated into the magnetosomes structure, they must be present in a very low content, lower than the resolution limit of the technique. On the other hand, the presence of small absorption peaks at the Co edge is indicating that some Co ions are also being incorporated into the magnetosome structure. In order to obtain more information about this, we have obtained the XMCD spectrum (*I*^+^ – *I*^–^) for MSGM + W at Fe L_2,3_-edge and compared it with the one expected for a Fe_3_O_4_ standard (see Figure 3b). As depicted, the XMCD spectrum within the Fe L_3_ spectral region consists of three main components related to the three different iron occupations of magnetite: Fe^2+^ and Fe^3+^ in octahedral places (Fe^2+^ Oh, Fe^3+^ Oh), and Fe^3+^ in tetrahedral places (Fe^3+^ Th). The sign of the magnetic dichroism for each component is defined by the relative orientation of its magnetic moment with respect the incoming beam direction. Negative XMCD correspond to Fe^2+^ Oh and Fe^3+^ Oh, which are aligned ferromagnetically, while the peak for Fe^3+^ Th showing a positive XMD is antiferromagnetically coupled to them. The comparison of the experimental data with the theoretical XMCD spectrum corresponding to magnetite,^60^ reveals a decrease in the relative Fe^2+^ Oh intensity. The relative intensity of the XMCD peaks corresponding to the 706.0 and 714.5 eV energy range allows estimating the site occupancy of the Fe cations (Fe^2+^ Oh/Fe^3+^ Th/Fe^3+^ Oh). We obtain a ratio of 0.84(5):0.95(5):1.00(6), very close to that found for unmodified magnetosomes of *M. gryphiswaldense* [1.00(4):1.02(5):0.96(5)] and that expected for stoichiometric magnetite (1:1:1).^48^ These results, together with the confirmed presence of Co^2+^ cations, suggest that in MSGM + W, Co^2+^ ions present in the medium partially substitute Fe^2+^ ions in octahedral positions (see Figure 3c). The total Co doping content is approximately 4–5%.

### Magnetic Characterization: Experiments and Simulations

Once we have structurally and morphologically characterized the AMB-1 bacteria grown in these three different media (MSGM + W, MSGM – W, and FSM), we have focused on studying how these media affect the magnetic response of the bacteria, which is especially relevant for their biomedical application.

**Figure 4:**
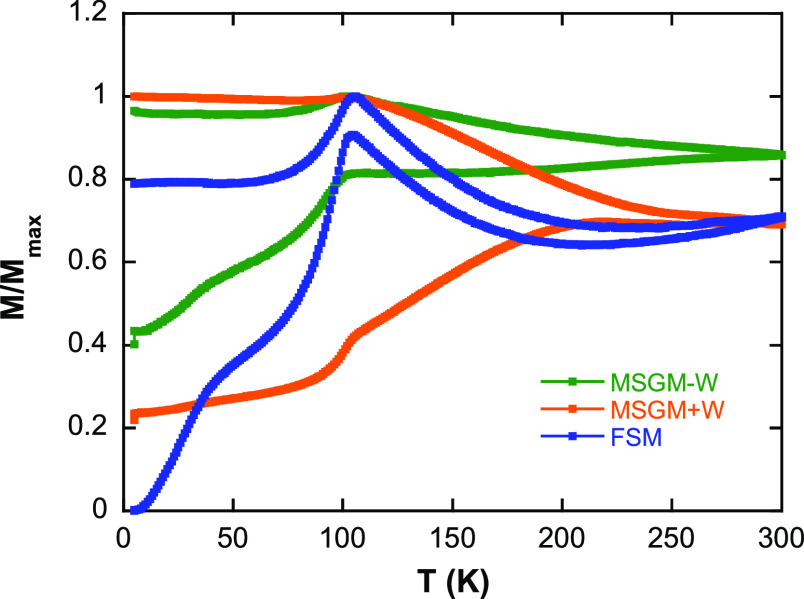
ZFC/FC curves measured at 5 mT of *M. magneticum* grown in MSGM – W, MSGM + W, and FSM.

First, we have measured the *M* versus *T* curves in ZFC/FC mode, as depicted in Figure 4. The SQUID measurements were carried out with the bacteria freeze-dried and randomly oriented inside a gelatin capsule. For all the samples, the ZFC/FC curves present a clear irreversibility in the range of temperatures analyzed. MSGM – W and FSM exhibit a qualitatively similar shape, resembling the one reported for other similar magnetosomes, such as in the case of *M. gryphiswaldense*.^18,61^ In both cases, there is a well-defined peak around ∼105 K, in the ZFC and FC curves, which corresponds to the Verwey transition.^62,63^ The fact that this transition is so abrupt and well-defined is indicative of the homogenous stoichiometry of magnetite in magnetosomes. The small differences between the ZFC/FC curves of these two samples could be ascribed to the small differences in the magnetosome size distribution and chain length reported before. However, for MSGM + W, the shape of the ZFC/FC curves is clearly different, and the Verwey transition is less sharp and only barely discernible at around 100 K. In addition, the broad shoulder around 40 K, which corresponds with the so-called low *T* transition in magnetite, is clearly absent for MSGM + W sample. This transition is again intrinsically related to the stoichiometry of magnetite.^62,64^ Therefore, all these results indicate that although the *M* versus *T* behavior for both MSGM – W and FSM is very similar, in the case of MSGM + W, the incorporation of the Co ions into the magnetosome structure, even in a small percentage, is affecting the magnetic response. For MSGM + W, the ZFC/FC curves strongly suggest an overall decrease of magnetite purity and/or increase of crystalline disorder. Similar changes have been in fact reported for other cobalt-doped magnetosomes.^48^

**Figure 5:**
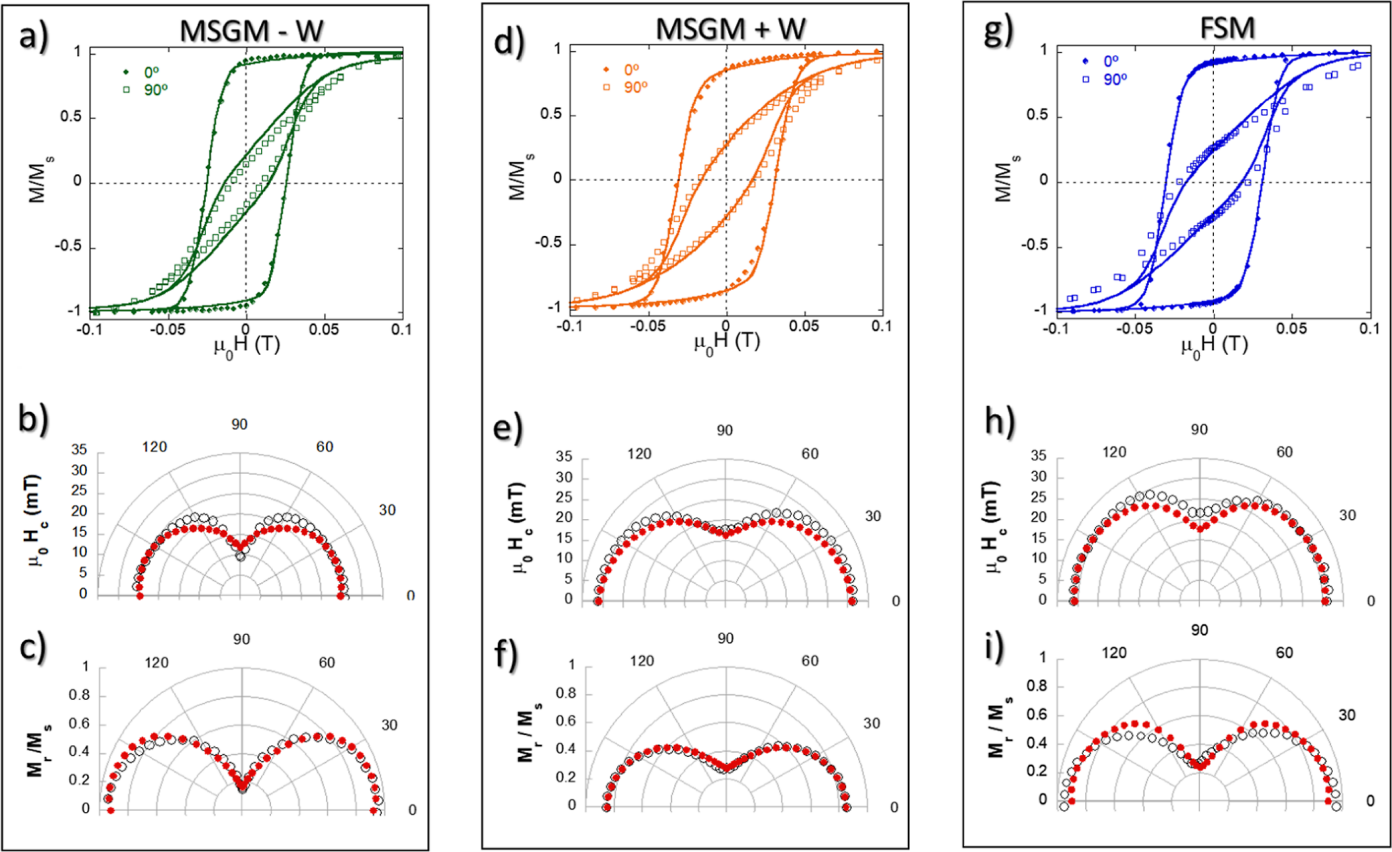
(a,d,g) Hysteresis loops of *M. magneticum* cultured in MGSM – W, MGSM + W, and FSM measured with the bacteria forming 0 and 90° with the aligning field. Polar plots of the (b,e,h) coercivity and (c,f,i) remanent magnetization. The solid lines (in the hysteresis loops) and red dots (in the polar plots) correspond to the values obtained from the simulations with a modified Stoner–Wohlfarth model, as described in the text.

After analyzing the magnetic response as a function of the temperature, we have studied the magnetization as a function of the magnetic field (*M* versus*H* hysteresis loops). These measurements were carried out using a VSM system, with maximum applied fields up to 1 T. In particular, we have measured the magnetic hysteresis loops as a function of the angle, Ω, that the aligned bacteria present with respect to the applied field, *H*_app_ (see Figure 1 for a schematic depiction). Figure 5 compares the *M* versus *H* loops obtained at 300 K for the oriented MSGM – W, MSGM + W, and FSM bacteria, forming angles between 0 and 180° with the applied magnetic field. MTB are highly anisotropic magnetic objects and their hysteresis loops measured at different angles depend strongly on the relative direction between the applied field and the alignment direction. As the angle between the chains of the bacteria and the applied magnetic field increases, the hysteresis loops become narrower due to the well-defined uniaxial anisotropy of the chain. However, we can also see that at 90°, there is still some remanent hysteresis for all three samples (Figure 5a,d,g). This indicates that magnetic chain arrangement cannot be described by a standard Stoner–Wohlfarth model of single uniaxial magnetic domains, since in that case anhysteretic loops would be expected at 90°. This disagreement can be attributed both to the likely presence of some small misalignment of the bacteria during the preparation of the samples and also to the tilting of the magnetic moments of the magnetosomes out of the chain axis due to the slight deformation of the truncated-octahedral shape of the magnetosomes, as demonstrated by Orue et al. in the species *M. gryphiswaldense*.^14,18^ In addition, if we compare the three samples, MSGM – W, MSGM + W, and FSM, we can observe some differences. Although the hysteresis loops of MSGM + W and FSM are similar, those of MSGM – W have smaller coercivity both at 0 and 90°.

These differences can be more easily analyzed if we compare the polar plots for the coercivity (Figure 5b,e,h) and normalized remanence (Figure 5c,f,i) of the 3 samples. As depicted, for MSGM – W, the value of the coercive field, μ_0_*H*_c_, at 0° is 25.7 mT, while at 90° it reaches a value of 15.9 mT. On the other hand, for MSGM + W, μ_0_*H*_c_ changes from 31.2 mT at 0° to 17.6 mT at 90°, while for FSM, it changes from 31.1 mT at 0° to 23.0 mT at 90°. This showcases the clearly higher μ_0_*H*_c_ value for FSM at 90°. On the other hand, the normalized remanence, *M*_r_/*M*_S,_ values for MSGM – W change from 0.92 at 0° to 0.17 at 90°, while for MSGM + W, they change from 0.85 at 0° to 0.27 at 90°. Finally, for FSM, *M*_r_/*M*_S_ goes from 0.96 at 0° to 0.29 at 90°. All these results indicate that at room temperature, the incorporation of Co ions into the magnetite core of the magnetosome gives rise to a modification of the coercivity and remanence for MSGM + W compared to MSGM – W. In addition, sample FSM, which in principle looked similar to MSGM – W in the magnetic response at low fields (see Figure 4), also exhibits some clear differences in the *M* versus *H* loops, with an enhanced coercivity and remanence.

To shed some light on this, we have carried out simulations using a modified Stoner–Wohlfarth model. A detailed description of this model has been reported elsewhere.^14,18^ Briefly, using this model, the equilibrium configuration of the magnetic moment of each magnetosome is calculated considering three contributions: (a) the magnetocrystalline anisotropy energy, *E*_C_; (b) the effective uniaxial anisotropy energy, *E*_uni_, due to the competition between the magnetosome shape anisotropy and the dipolar interactions between magnetosomes inside the chain; and (c) the Zeeman energy term, *E*_Z_. In spherical coordinates, considering the ⟨100⟩ crystallographic directions of magnetite as the reference system, the total energy density is given by
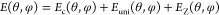
1where
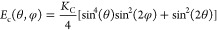
2
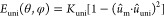
3
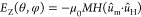
4being θ and φ the polar and azimuthal angles of the magnetic moment of each magnetosome, respectively; *K*_c_ is the magnetocrystalline anisotropy constant; *K*_uni_ is the effective uniaxial anisotropy constant, coming from shape anisotropy and the dipolar interaction of the magnetosomes in the chain;  is the magnetic moment unit vector;  is the uniaxial anisotropy unit vector; and  is the external magnetic field unit vector. As shown in previous works with *M. gryphiswaldense*,  is tilted ∼20° out of the ⟨111⟩ chain axis direction^14,18^ due to the slight deformation of the cube-octahedral shape of the magnetosomes. Considering the similar morphology of the magnetosomes in both species, in this work, we also consider a possible deviation of  out of the ⟨111⟩ chain axis direction for *M. magneticum*. Misalignments of the chains with respect to the aligning field occurring during sample preparation have been also considered by including a Gaussian angular distribution of the chain axes. We have tested three angular distributions around the chain axis: 15, 20, and 25°, obtaining the best results for an angular distribution of 20°. Using these considerations, the *M* versus *H* loops at different angles have been simulated using a dynamical approach described elsewhere.^18,65^*K*_c_ and *K*_uni_ have been adjusted to attain the best match between experimental and simulated *M* versus *H* loops.

As depicted in Figure 5a,d,g, we have been able to simulate quite accurately the hysteresis loops of MSGM – W, MSGM + W, and FSM bacteria using this model. The accuracy of the fittings is even more evident in the polar plots of the reduced remanent magnetization and coercivity. In the three cases, a tilting of 20° for the uniaxial anisotropy of the magnetosome () with respect to the chain axis has proven to give the best results. By setting this angle, the effective easy axis is found to lie 15° out of the chain axis. This suggests that the magnetosomes from *M. magneticum* also present a deformation comparable to that of *M. gryphiswaldense*, although high-resolution electron cryotomography or similar images would be needed to ascertain this.^14^ From the simulations, we obtain different values for *K*_c_ and *K*_uni_, as shown in Table 3. To take into account the size dispersion existing in the magnetosomes, a Gaussian distribution for *K*_uni_ has been considered. The value of *K*_c_ for both MSGM – W and FSM is very similar (−11 kJ/m^3^). This was also the value obtained for *M. gryphiswaldense* and is the value reported for bulk magnetite crystal (*K*_c_ = −10/–11 kJ/m^3^).^31^ However, the value of *K*_c_ radically changes in the case of MSGM + W, reaching a positive value of +3 kJ/m^3^. This great increase of cubic magnetocrystalline anisotropy is a strong confirmation of the incorporation of cobalt ions to the spinel structure of magnetite,^48^ as suggested by the XAS and XMCD data. On the other hand, both MSGM + W and FSM, present slightly higher values for *K*_uni_ (∼16 kJ/m^3^) than MSGM – W (∼12 kJ/m^3^), whose value is similar to the one reported for *M. gryphiswaldense* bacteria (∼12 kJ/m^3^).^14^ This increase in the uniaxial anisotropy observed for MSGM + W and FSM could be related either to changes in the morphology of the magnetosomes that would affect the shape anisotropy or to differences in the chain configuration affecting the dipolar interactions in the chain, as we saw before (see Table 1). Nevertheless, a change in the morphology of the magnetosomes should not be discarded. Bacteria have been shown to distort the morphology of magnetosomes to more easily accommodate the chain within the bacteria morphology.^14,18^

**Table 3:** Parameters Obtained from Simulations of *M* versus *H* Loops for Oriented MSGM – W, MSGM + W, and FSM Bacteria (Figure 5)^a^

	*K*_c_(kJ/m^3^)	*K*_uni_(kJ/m^3^)	tilting (deg)
MSGM – W	–10	12(4)	15
MSGM + W	3	16(4)	15
FSM	–10	16(4)	15

a *K*_c_ and *K*_uni_ are the magnetocrystalline and uniaxial anisotropies, respectively. In *K*_uni_, the parentheses correspond to the width of the Gaussian angular distribution of the chain axes, as explained in the text. The tilting corresponds to the angle between the effective easy axis of the magnetosomes and the chain axis, given by the direction ⟨111⟩.

The obtained magnetic results clearly showcase that using different media allow us to modify the magnetic response of the magnetosomes. This can also serve as a warning for groups working with this or other species of MTB grown in media that contain Co or other transition metals, since there is a real chance that some of these elements are inadvertently incorporated into the magnetosome structure, thereby modifying the final magnetic response of the magnetosomes.

### Magnetic Hyperthermia Characterization

**Figure 6:**
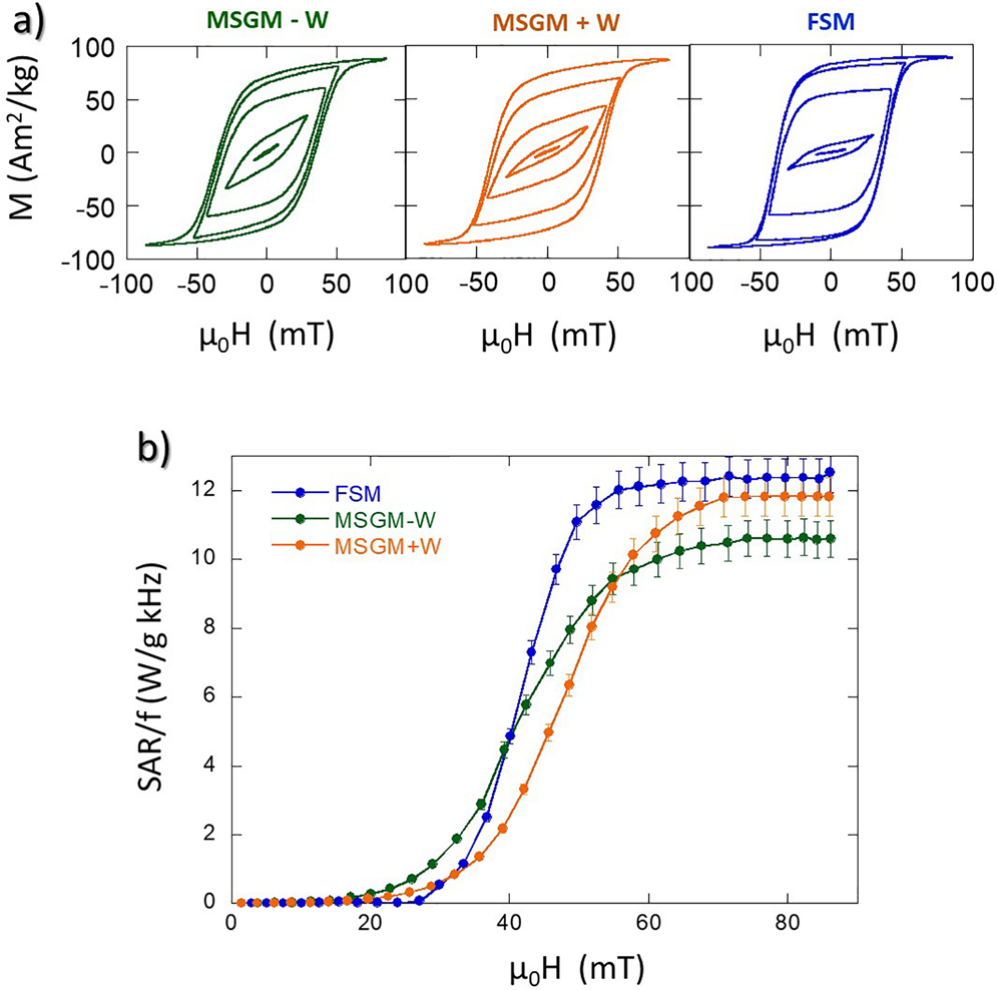
(a) AC hysteresis loops of MSGM – W, MSGM + W, and FSM bacteria dispersed in water, measured at an AC magnetic field with a frequency of 149 kHz and a maximum amplitude of 80 mT. (b) SAR normalized by the frequency (SAR/*f*) as a function of the amplitude of the AC magnetic field at a frequency of 149 kHz for MSGM – W, MSGM + W, and FSM bacteria.

Our results show that the culture media used for bacterial growth affect their magnetic behavior due to the important changes they cause in the magnetic anisotropy of the magnetosome chains. This matter should also affect the magnetic hyperthermic response of bacteria. To address this point, the heating efficiency of bacteria has been measured by AC magnetometry, see Figure 6.

As shown in Figure 6a, with increasing magnetic field, the AC loops evolve from the typical lancet shape of a minor loop to a rectangular loop, the shape expected for bacteria aligned parallel to the magnetic field. This suggests that bacteria dispersed in water are aligned in the direction of the AC field, as previously observed in ref,^11^ giving rise to an optimum magnetic response for hyperthermia, with high remanence and coercivity. Although the evolution is similar for the three samples, some differences can be observed in the shape of the AC loops both at low and high fields. These differences can be more easily analyzed if we study the heating efficiency or SAR as a function of the applied field. Figure 6b displays the evolution of the SAR values normalized by the frequency, *f*, as a function of the applied magnetic field amplitude, *μ*_0_*H*, for MSGM – W, MSGM + W, FSM bacteria dispersed in water. The SAR values (in W/g) were directly obtained from the area (*A*) of the AC hysteresis loops, according to the equation^66^
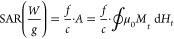
5*M*_*t*_ being the instantaneous magnetization at time *t*, *H*_t_ the sinusoidal magnetic field of frequency *f* at time *t*, and *c* the magnetite weight concentration in the dispersing medium. The integration is done over a period of the oscillating magnetic field, *T* = 2π/*f*.

The evolution of the SAR/*f* curves as a function of the applied field follows a similar qualitative trend for the three samples. SAR/*f* values are nearly negligible below a certain threshold field, around 30–35 mT. In this low-field region, bacteria grown in MSGM – W provide the best heating results. This can be directly related to the lower *K*_uni_ of these bacteria compared to the ones grown in MSGM + W and FSM, as indicated in Table 3, which facilitates the orientation of the magnetic moments of the magnetosomes with the applied field, and thereby gives rise to AC loops with larger area at low-applied fields, as has been explained before.^27,65,67^ Above that field, the SAR/*f* curves increase very rapidly, with a slope of 0.65, 0.45, and 0.40 W g^–1^ kHz^–1^ mT^–1^ for FSM, MSGM + W, and MSGM – W bacteria, respectively. The faster the increase of the SAR/*f* versus H curve, the easier for the magnetic moments to align along the direction of the applied magnetic field. As shown by Gandia et al., the alignment of the magnetic moments with respect to the field directions is a combined effect of both the intrinsic rotation of the magnetic moments of the magnetosomes inside the MTB and the possible physical rotation of the whole MTB.^11^ Hence, a possible explanation for the largest SAR/*f* versus H change rate for FSM as compared to MSGM – W and MSGM + W could be that FSM bacteria, having longer subchains, can align more easily with the magnetic field due to the higher magnetic torque. Nevertheless, there could be some other effects playing a small role, like the differences in magnetite concentration for the 3 samples (0.42, 0.63, and 0.17 mg Fe_3_O_4_ mL^–1^ for MSGM – W, MSGM + W, and FSM bacteria, respectively). In this respect, one must take into account that the differences in concentration are not going to greatly affect the heating efficiency of the MTB. Contrary to what happens in isolated nanoparticles, where concentration can play a key role since higher concentration implies stronger dipolar interactions and higher tendency to agglomeration, in the case of the MTB, the magnetosomes are surrounded by the bacterial body, and therefore, changing the concentration of bacteria does not affect the interactions of the magnetosomes inside each bacteria or increase the tendency to agglomeration. Finally, at higher magnetic fields, μ_0_*H* ≥ 60 mT, SAR/*f* reaches a saturation value ∼12 W g^–1^ kHz^–1^ for FSM and MSGM + W, and ∼10.5 W g^–1^ kHz^–1^ for MSGM – W bacteria. The higher SAR/*f* obtained for FSM and MSGM + W compared to MSGM – W can be again related to their higher *K*_uni_. These results show that the heating efficiency of *M. magneticum* can be tuned by changing the culture medium: below 35 mT, the best heating results are provided by MSGM – W; but above 60 mT, both FSM and MSGM + W provide the highest SAR values.

Under the currently most accepted safety limit for clinical hyperthermia (i.e., Hergt criterion, *H* × *f* ≤ 5.0 × 10^9^ A m^–1^ s^–1^),^68^ for a frequency *f* = 150 kHz, a field amplitude not higher than ∼42 mT should be applied. However, latest in vivo results have shown that increasing this limit up to *H* × *f* ≤ 9.59 × 10^9^ A m^–1^ s^–1^^69^ seems in principle to be safe, although further research is needed. Therefore, under this latest restriction, all the field amplitudes applied in this study can be considered within the safety limit. Taking this into account, it is clear that FSM and MSGM + W bacteria are the best heating mediators among the three bacteria studied in this work; and in particular, FSM bacteria when low fields have to be used or to comply with lower or more demanding safety limits.

## Conclusions

We have carried out a systematic study on the magnetic properties and heating efficiency of *M. magneticum* AMB-1 as a function of the culture medium. Three different culture media have been employed: MSGM – W, MSGM + W, and FSM. The study has revealed that all culture media can be employed to grow *M. magneticum* AMB-1 with fully formed chains of magnetosomes but with different magnetic response. The conventionally used MSGM + W for culturing *M. magneticum* AMB-1 originates a doping of the Fe_3_O_4_ magnetosomes with 4–5% Co^2+^ ions, which are incorporated into Oh sites. This changes the magnetic response of the magnetosomes, originating a pronounced increase in magnetocrystalline anisotropy, magnetic coercivity, and remanence. FSM presents as an interesting alternative to grow this species. The bacteria grown in this medium present high uniaxial anisotropy, which results in a high heating efficiency without having to use the standard MSGM + W. For fields μ_0_*H* ≥ 60 mT, a maximum SAR_max_/*f* ≈ 12 W g^–1^ kHz^–1^ is obtained both for MSGM + W and FSM, which is even higher than some of the best values reported in high-quality chemically synthesized magnetite NPs (SAR_max_/*f* ≈ 6 W g^–1^ kHz^–1^).^70^

Therefore, these results put emphasis on the importance of the culture medium to control and tune the magnetic response of the MTB, opening the door to the possibility of selecting the most appropriate culture medium, according to the specific biomedical application, as we have seen in the particular case of magnetic hyperthermia.
